# The impact of COVID-19 on mental health and posttraumatic growth of Korean college students: a mixed method study examining the moderating role of coping flexibility and sense of community

**DOI:** 10.3389/fpsyg.2023.1200570

**Published:** 2023-07-20

**Authors:** Jae-Chang Sim, Sun-Young Im

**Affiliations:** Department of Psychology, Hallym University, Chuncheon-si, Gangwon-do, Republic of Korea

**Keywords:** COVID-19 pandemic, mental health, posttraumatic growth, coping flexibility, sense of community, moderation, qualitative study

## Abstract

**Introduction:**

In the context of the COVID-19 pandemic, which has led to complex psychological problems, it is important to examine the effect of coping flexibility and sense of community, because relying solely on specific coping strategies is ineffective, and the pandemic necessitates social cooperation.

**Methods:**

This study was divided into two parts. The first study used a quantitative research method(i.e., structural equation modeling) to test if coping flexibility and sense of community moderated the impact of COVID-19-related concerns on mental health (i.e., depression and anxiety) and posttraumatic growth among Korean college students. The second study used a qualitative research method for an in-depth examination of how Korean college students coped with the COVID-19 pandemic and if they achieved any positive change or growth. Given that the COVID-19 pandemic represents a situation distinct from what people have previously encountered, Study II was designed to examine the experiences of individuals during this exceptional period.

**Results:**

In the first study (Study I), coping flexibility was found to increase the impact of COVID-19-related concerns and difficulties on depression and anxiety. Conversely, a sense of community reduced the consequences of these overwhelming worries on depression and anxiety, while also expanding the impact of COVID-19-related disorders on posttraumatic growth. In the second study (Study II), the findings showed that the participants experienced various psychological consequences, including depression and anxiety, and distress in other aspects of their life, including disruptions in interpersonal relationships and college life. Nonetheless, the participants made efforts to cope with such difficulties and overcome the challenges together with the community. In fact, the pandemic improved their coping skills and expanded their value system and worldview.

**Conclusion:**

The study findings suggest that given the unique situation presented by the COVID-19 pandemic, a sense of community protected the mental well-being of Korean college students and facilitated their growth. This study emphasizes the necessity of promoting SOC to effectively cope with disaster situations.

## 1. Introduction

The COVID-19 pandemic has had a profound socioeconomic impact globally and on the community at large. Previous epidemics, such as the Middle East respiratory syndrome (MERS) or severe acute respiratory syndrome (SARS), caused various psychological problems, including the fear of being infected, feeling of isolation, suffering owing to the associated social stigma when infected, anxiety, and depression (Jeong et al., 2016; Lee et al., 2016; Ha and Ban, 2017; Kim et al., 2018). Thus, COVID-19 has emerged as a serious global threat due to its explosive infection potential, surpassing both the MERS and SARS epidemics (Petrosillo et al., 2020).

How do people cope with the COVID-19 pandemic? People deal with the challenges associated with it in a variety of ways (Frey et al., 2021), but not all of them are successful (Cheng, 2003). Some studies argue that coping flexibility (*CF*), that is, the ability to respond flexibly to situations (e.g., COVID-19), is more effective in coping with stress than the use of specific coping (Cheng, 2003). It is also argued that a sense of community (SOC), such as a bond with the community, is important in overcoming the difficulties, given that the COVID-19 pandemic affects the state and society as a whole. For example, an SOC was expected to play an important role in Korea’s COVID-19 quarantine success cases. There is also the view that people will grow by overcoming the challenges of the COVID-19 pandemic (Cui et al., 2021), which is worth verifying. This study focuses on whether *CF* and SOC can actually protect people from the impacts of the COVID-19 pandemic, and whether people can grow psychologically from these impacts.

### 1.1. The impact of COVID-19

The pandemic not only restricted normal daily life, but also had a psychological impact. During the pandemic, people wore a mask when going out, mass gatherings were legally restricted, and people were reluctant to hold personal gatherings due to the fear of infection. Moreover, people experienced various psychological problems, including anxiety, depression, fear, anger, and social loneliness (Lee et al., 2020; Wang et al., 2020). Recently, the media coined new terms such as “Corona depression” or “Corona blues,” indicating increased public interest in the negative impact of COVID-19. Lee et al. (2020) categorized approximately 30 and 50% of their study participants into depressed and anxiety groups, respectively. Moreover, a meta-analysis on the association between COVID-19 lockdown and mental health conducted by Prati and Mancini (2021) reported that the lockdown period during COVID-19 had a psychological impact causing anxiety and depression.

Many studies have showed that the number of patients reporting depression during the COVID-19 pandemic was high compared to that in previous epidemics (Korean Ministry of Health and Welfare, 2021, May 6; Medigate News, 2021, January 28). According to the Korean Ministry of Health and Welfare (2021, May 6), depression levels had more than doubled among people in 2021 as compared to 2018; in particular, individuals in the age groups of 20–29 years and 30–39 years showed the highest risk of depression. Increased depressive symptoms among relatively younger generations could be interpreted as being important. For most Koreans students, the age of 20–29 years represents their college years, while this is also the period of entry into early adulthood when individuals mature and develop self-identity, gain emotional stability, and form congenial relationships with others (Jo et al., 2016). Erikson (1963) emphasized the psychosocial aspects of personality development and theorized that early adulthood covers the period when an individual develops his/her personality by forming close relationships. However, considering that the stay-at-home COVID-19 restrictions disrupted such meeting grounds, preventing people from forming close relationships, this can be a major crisis for psychosocial development.

### 1.2. Coping flexibly to adapt to COVID-19

The aspect about coping strategies being useful during the COVID-19 pandemic needs to be addressed. Previous studies identified various coping responses among people during the COVID-19 pandemic (Salin et al., 2020; Frey et al., 2021). Frey et al. (2021) found that patients with ovarian cancer were using various coping strategies during the COVID-19 pandemic, including seeking emotional support, self-care, hobbies, positive reframing, and self-blame. The types of coping methods were categorized as follows: problem-focused versus emotion-focused coping; approach versus avoidance coping (Lazarus and Folkman, 1984); and problem-solving versus social support-seeking versus avoidance coping (Amirkhan, 1990). According to previous studies, problem-focused coping showed higher correlations with adaptive indicators such as psychological well-being than did emotion-focused coping (Krantz and Moos, 1987; Aldwin, 1993; Littrell and Beck, 2001). Avoidance coping, such as denial, self-blame, and behavioral disengagement, was associated with a lower sense of well-being, whereas approach coping, such as emotional support and positive reframing, was associated with a strong sense of well-being (Dawson and Golijani-Moghaddam, 2020).

However, it is questionable if certain types of everyday coping situations have been as effective during the pandemic, which is a serious crisis that humans have faced for the first time; moreover, the pandemic is expected to show other complex and unique characteristics than everyday stress from the past. For example, the COVID-19 pandemic requires coping and cooperation at national and global levels, which are difficult to control at the individual level. While social activities have been severely restricted, advances in science and technology have led to the need to adapt to social activities through non-face-to-face methods, thus increasing family time. This has led to high parenting stress or conflict situations. Forsythe and Compas (1987) demonstrated that problem-focused coping in situations that can be controlled and emotion-focused coping in situations that cannot be controlled may be more adaptive. In the same context, several studies have supported that cognitive reappraisal (Levenson et al., 1989; Troy et al., 2013) is more effective in uncontrollable situations than in controllable situations, and problem-focused coping (Kim et al., 1997; Cheng et al., 1999) is more effective for managing stress or anxiety in controllable situations than in uncontrollable situations. In summary, instead of using the same coping methods across the board for all situations, using flexible coping mechanisms, based on the characteristics of each situation, could be important for adaptation (Han et al., 2001; Kato, 2001) during the COVID-19 pandemic.

The concept that a specific coping mechanism may not be effective in all situations has been conceptualized as *CF*. In fact, *CF* is considered to be a good match between a coping strategy and stressful event (Linvile and Clark, 1989; Aldwin, 1994); individuals with high *CF* have the ability to cope effectively as they are expected to assess the situation and adapt to it, as observed during the pandemic. *CF* is associated with the ability to accept ambiguity (Cheng, 2003), wherein high *CF* can reduce uncertainty and fear, as witnessed during the COVID-19 pandemic, and facilitate appropriate coping. Cheng et al. (2021) identified that individuals with higher *CF* experienced lower anxiety levels about personal health, other behaviors such as hoarding, and lower levels of depressive symptoms. Reportedly, *CF* is also associated with various positive mental health indicators. For example, *CF* had a positive association with life satisfaction (Ng et al., 2014), and it moderated the association between trauma exposure and post-traumatic stress disorder (PTSD) symptoms (Bonanno et al., 2011). Moreover, *CF* acted as a protective factor for PTSD and depressive symptoms (Park et al., 2015).

### 1.3. Relying on the community to adapt to COVID-19

Meanwhile, Korea’s response measures against COVID-19 have collectively been referred to as “K-Quarantine,” and this has attracted global attention (Korea Policy Briefing, 2020, December 22). In June 2020, in an interview posted on YouTube by the Ministry of Foreign Affairs, Harvard University professor Michael J. Sandel mentioned that one of the reasons Korea was able to achieve successful quarantine, in comparison to neighboring countries, was the broader meaning of SOC and social solidarity, emphasizing SOC as Korea’s successful response to COVID-19.

Indeed, SOC is considered an important element in public health (Ko et al., 2021), receiving attention as a contextual factor that enhances individuals’ well-being and protects them from psychological suffering in stressful situations (Gattino et al., 2013; Ryu, 2014). McMillan and Chavis (1986) defined SOC as experiencing a sense of belonging or membership, influence, integration and fulfillment of needs, and shared emotional relationships within a community. Many previous studies have reported that SOC not only reduces stress or depression, but is also associated with various positive psychological indicators, such as happiness, positive stress coping, quality of life, psychological health, subjective happiness, satisfaction with school or military life, and lower stress levels and depression (Greenfield and Marks, 2010; Gattino et al., 2013; Huang and Wong, 2013; Kang and Jang, 2013; Rollero et al., 2014; Ryu, 2014; Cho and Kim, 2016; Kim and Kim, 2017; Lee, 2018; Baek et al., 2019). Mannarini et al. (2021) showed that SOC alleviated the impact of COVID-19 across various life domains, including family relationships, social life, leisure, income, physical and mental health, while positively predicting psychological well-being. Moreover, SOC may be associated with such positive outcomes by promoting harmony and cooperation (Prezza et al., 2008) and reducing social loneliness or the sense of isolation (Al-Omoush et al., 2020).

### 1.4. Psychological growth from COVID-19

Today, while the ongoing COVID-19 pandemic has undoubtably had a negative impact on everyone, this tragic pandemic has also provided opportunities for growth and positive change at both personal and societal levels (Veteran Family Wellness Center, n.d.). In other words, difficulties experienced during COVID-19 have helped people become mature (Zhou et al., 2020; Cui et al., 2021). During the pandemic, people may have realized and understood several things, including social responsibility, cooperative behavior, coping with the situation, and overcoming the sense of isolation. Trauma or stress events can positively predict subsequent growth (Levi and Bachar, 2019). Posttraumatic growth (PTG) is a term that represents the subjective experience of an individual reporting positive psychological changes after combating a traumatic event (e.g., COVID-19 pandemic), indicating a positive growth experience (Maercker and Zoellner, 2004).

*CF* and SOC are expected to facilitate PTG among Koreans. Cohen and Katz (2015) reported that individuals’ *CF* showed a significant correlation with their PTG. A study by Kunz et al. (2018) also reported that *CF* not only helped individuals recover by buffering negative psychological indicators, but it also acted as a predictor of PTG. In other words, *CF* not only alleviated individuals’ negative psychological responses to the COVID-19 pandemic, but also acted as a factor that can promote positive change. Moreover, PTG is expected to enhance SOC (Prati and Pietrantoni, 2009). Females with prosocial behaviors, such as in-person volunteering, were reported to have a higher level of PTG than females who did not participate in volunteering or contributed only toward online volunteering (Freedle and Oliveira, 2021). Han et al. (2020) identified that the survivors of the Sewol ferry disaster in Korea had formed a strong support system that enabled them to empathize with each other as a community; this was an important factor that helped survivors not only recover from the suffering, but also provided them with psychological growth. Several other studies also support the view that interventional programs for facilitating SOC have a positive effect on post-traumatic recovery and growth (Sautter et al., 2011; Monk et al., 2017; Norman et al., 2020).

### 1.5. Purpose

In summary, using only certain types of coping during COVID-19 situations that cause complex psychological problems may not be useful for psychological adaptation and mental health. What is important during this time is the ability to use *CF*. Moreover, SOC could help, given that the situation caused by the pandemic is beyond one’s control and requires social cooperation. *CF* and SOC can also encourage people to seize growth opportunities during COVID-19. However, if the pandemic involves special circumstances that differ from daily life, it is not enough to be understood simply by statistical verification. For instance, PTG involves profound changes experienced by individuals facing significant life crises, and to understand and describe it in detail, qualitative research is necessary. Additionally, qualitative exploration would be useful in understanding the mechanisms underlying *CF* and SOC.

This study employed a mixed method approach. Study I was designed to test the moderating effects of *CF* and SOC on the impact of COVID-19-related concerns and difficulties on depression, anxiety, and PTG among Korean college students. The main hypotheses of Study I were that (1) COVID-19-related concerns and difficulties will increase depression, anxiety, and PTG; and (2) *CF* and SOC will reduce the effect on depression and anxiety, and increase the effect on PTG. These hypotheses were tested by structural equation modeling. For a more sophisticated investigation of the findings, Study II was designed as a qualitative research using in-depth interviews to specifically examine, during the COVID-19 pandemic, what negative experiences (pain) and positive changes (growth) Korean college students experienced and what specific roles *CF* and SOC played in those experiences.

## 2. Study 1

### 2.1. Materials and methods

#### 2.1.1. Participants

A total of 296 Korean college students participated in the online survey, having first given their informed consent. The survey was distributed to various internet communities used by Korean college students. Subsequently, the number of participants was reduced to 277 after excluding students who stated that they did not experience the challenges of COVID-19 pandemic during college life and those who participated in the survey more than once. Among the 277 participants, three participants who used just one number as the response in three or more scales, and three participants who were rejected as outliers based on *p* < 0.001 found in Mahalanobis’s distance (*D*^2^), were excluded from the total score for all scales (Kline, 2015). Consequently, data from a total of 271 participants were used in the final analysis. A realistic ratio of the sample size to produce stable results in statistical modeling is 5:1 (Bentler and Chou, 1987). The number of parameters to be estimated in the model tested in this study was 53 (13 factor loadings, 19 measurement error variances, 6 latent variable variances, and 15 path coefficients or covariances) in each measurement and structural model; thus, 271 comprised an appropriate sample size for the model tests. All participants reported their sex, age, socioeconomic status, religion, and primary residence (Table 1). The mean participant age was 21.41 years with a standard deviation (SD) of 2.27; minimum and maximum ages were 18 and 31 years, respectively.

**Table 1:** Demographic variables of Study I participants.

Demographic variables		Count	Rate (%)
Total		271	100.00
Sex	Male	62	22.88
	Female	209	77.12
Vaccinated	Yes	150	55.35
	No	121	44.65
Economic level	High	22	8.12
	Middle	203	74.91
	Low	46	16.97
Religious	Religious	85	31.37
	Non-religious	186	68.63

#### 2.1.2. Measures

##### 2.1.2.1. COVID-19-related concerns and difficulties

Concerns and difficulties that people experienced during the COVID-19 pandemic were measured using two questionnaires: (i) a questionnaire used by Lee et al. (2020) to measure difficulties during the COVID-19 pandemic arising out of fear and non-dailiness, and (ii) the questionnaire Son et al. (2020) used for a COVID-19-related survey. All items were rated on a five-point Likert scale. An exploratory factor analysis on the “COVID-19-related concerns and difficulties” scale constructed for the present study confirmed that the items could be categorized into two factors of “concerns” and “difficulties.” The scale consisted of a total of 19 items and the internal consistency of the scale (Cronbach’s *α*) was 0.86.

##### 2.1.2.2. Depression

Depression was measured using the Korean version of the Center for Epidemiologic Studies Depression Scale (CES-D) scale, originally developed by Radloff (1977) and subsequently standardized and integrated by Chon et al. (2001). The integrated Korean version of the CES-D (Chon et al., 2001) is a 20-item scale, and Chon et al. (2001) study reported a four-factor structure of CES-D. Each item in the CES-D was rated on a four-point Likert scale, with higher scores indicating greater depressive symptoms. The internal consistency of the entire scale (Cronbach’s *α*) was 0.91 in Chon et al. (2001) study and 0.92 in the present study.

##### 2.1.2.3. Anxiety

Anxiety was measured using the Generalized Anxiety Disorder-7 (GAD-7) scale developed by Spitzer et al. (2006). The GAD-7 is a seven-item scale that has a one-factor structure. Each participant rated the anxiety symptoms they had experienced in the past 2 weeks on a four-point Likert scale. The present study used the translated Korean version of the scale provided by the original developers through the official website.^1^ The internal consistency of the scale (Cronbach’s *α*) was 0.89 in Spitzer et al. (2006) study and 0.91 in the present study.

##### 2.1.2.4. Posttraumatic growth

PTG was measured using the Korean version of the Posttraumatic Growth Inventory Expanded scale (PTGI-X). Originally developed as the PTGI scale (Tedeschi and Calhoun, 1996), it was subsequently revised with the addition of existential items by Tedeschi et al. (2017), which was adapted into Korean and validated by Kim et al. (2020). The participants rated the positive changes they experienced during the COVID-19 pandemic on a six-point scale (0 = I did not experience this change, 1 = I experienced this change to a very small degree, 2 = I experienced this change to a small degree, 3 = I experienced this change to a moderate degree, 4 = I experienced this change to a great degree, and 5 = I experienced this change to a very great degree). The Korean version of the PTGI-X has four domains: change in self-perception, increase in interpersonal depth, increase in spiritual and existential depth, and discovery of new possibilities. The internal consistency of the entire scale (Cronbach’s *α*) was 0.90 in Kim et al. (2020) study and 0.96 in the present study.

##### 2.1.2.5. Coping flexibility

*CF* was measured using the Korean version of the Coping Flexibility Questionnaire (COFLEX), originally developed by Vriezekolk et al. (2012) and subsequently translated into Korean and validated by Song and You (2018). The Korean version of COFLEX has 12 items, one less than the original version, and each item is rated on a four-point Likert scale with higher scores indicating higher *CF*. The Korean version of COFLEX has three sub-factors: perceived coping resources, assessment of coping behavior, and flexibility in coping behavior. The internal consistency of the entire scale (Cronbach’s *α*) was 0.85 in the study by Song and You (2018) and the present study.

##### 2.1.2.6. Sense of community

SOC was measured using the Brief Sense of Community Scale (BSCS), which was originally developed as the Sense of Community Index (SCI) based on McMillan and Chavis (1986)’s theory by Perkins et al. (1990) and subsequently condensed and validated by Peterson et al. (2008). The BSCS is an eight8-item scale that includes four domains: needs fulfillment, membership, influence, and emotional connection. In this study, the BSCS items were translated into Korean by researchers and the term “neighborhood” was revised to “community” or “country.” Each item was rated on a five-point Likert scale, with higher scores indicating a stronger SOC. The internal consistency of the entire scale (Cronbach’s *α*) was 0.89 in this study.

#### 2.1.3. Analysis

All statistical analyses were performed using the R version 4.0.5. First, descriptive statistics of the major scales were derived, and the Pearson product–moment correlation analysis was performed to investigate the correlations between the major scales. Subsequently, differences in COVID-19-related concerns and difficulties according to demographic variables were tested. Lastly, structural equation modeling (SEM) was performed to test the moderating effects of *CF* and SOC on the impact of COVID-19-related concerns and difficulties on depression, anxiety, and PTG (Marsh et al., 2004). COVID-19-related concerns and difficulties, *CF*, SOC, and anxiety were parceled by a factorial algorithm (or the single-factor analysis parceling method) (Landis et al., 2000), while depression and PTG were parceled by the content-based parceling approach.

### 2.2. Results

#### 2.2.1. Descriptive statistics and results of correlation analysis

Table 2 shows the descriptive statistics and correlation analysis results for all the scales. The skewness and kurtosis of all scales met the criteria for multivariate normality (skewness <2 and kurtosis <7) proposed by Curran et al. (1996). Since the multivariate normality was satisfied, the maximum likelihood method was used for parameter estimation of SEM. The correlation analysis results showed that COVID-19-related concerns and difficulties had significant positive correlations with depression (*r* = 0.40, *p* < 0.001), anxiety (*r* = 0.48, *p* < 0.001), and PTG (*r* = 0.26, *p* < 0.001). Depression showed a strong positive correlation with anxiety (*r* = 0.83, *p* < 0.001) and a weak negative correlation with SOC (*r* = −0.17, *p* < 0.005). PTG showed significant positive correlations with *CF* (*r* = 0.41, *p* < 0.001) and SOC (*r* = 0.53, *p* < 0.001).

**Table 2:** Description and correlations of measures.

	COV	CES-D	GAD-7	PTGI-X	COFLEX	BSCS
**Correlation**						
COV	1					
CES-D	0.40^***^	1				
GAD-7	0.48^***^	0.83^***^	1			
PTGI-X	0.26^***^	−0.05	0.07	1		
COFLEX	0.12	−0.06	0.02	0.41^***^	1	
BSCS	0.11	−0.17^**^	−0.07	0.53^***^	0.36^***^	1
**Description**						
*M*	47.27	22.29	6.99	58.85	24.06	18.76
*SD*	11.90	12.29	5.55	27.35	5.70	6.09
*Min*	13	1	0	2	10	0
*Max*	74	56	21	122	36	32
Skewness	−0.12	0.56	0.73	0.07	0.06	−0.31
Kurtosis	−0.17	−0.51	−0.33	−0.41	−0.63	0.18

^**^p < 0.01, ^***^p < 0.001. COV, scale for concern/difficulty during COVID-19, CES-D, integrated adaptation of CES-D in Korean; GAD-7, generalized anxiety disorder-7 in Korean; PTGI-X, Posttraumatic growth inventory - expanded in Korean; COFLEX, coping flexibility questionnaire in Korean; BSCS, brief sense of community scale.

#### 2.2.2. Differences according to demographic variables

Differences in the level of COVID-19-related concerns and difficulties according to sex, vaccination status, and religious status were assessed by a *t*-test. The total scores for COVID-19-related concerns and difficulties according to the religious status did not satisfy the assumption of homoscedasticity; thus, Welch’s t-test was performed when testing the effects of religious status. First, there were significant differences in COVID-19-related concerns and difficulties according to sex [*t*(269) = −2.433, *p* = 0.016], and males (*M* = 44.06, *SD* = 11.61) showed a higher mean score than females (*M* = 48.22, *SD* = 11.85). The effect size of sex was found to be weak (*d* = 0.352). However, significant differences were not observed in COVID-19-related concerns and difficulties according to vaccination status [*t*(269) = −0.367, *p* = 0.714] and religious status [*t*(135.14) = −0.761, *p* = 0.448].

#### 2.2.3. Impact on depression

**Figure 1:**
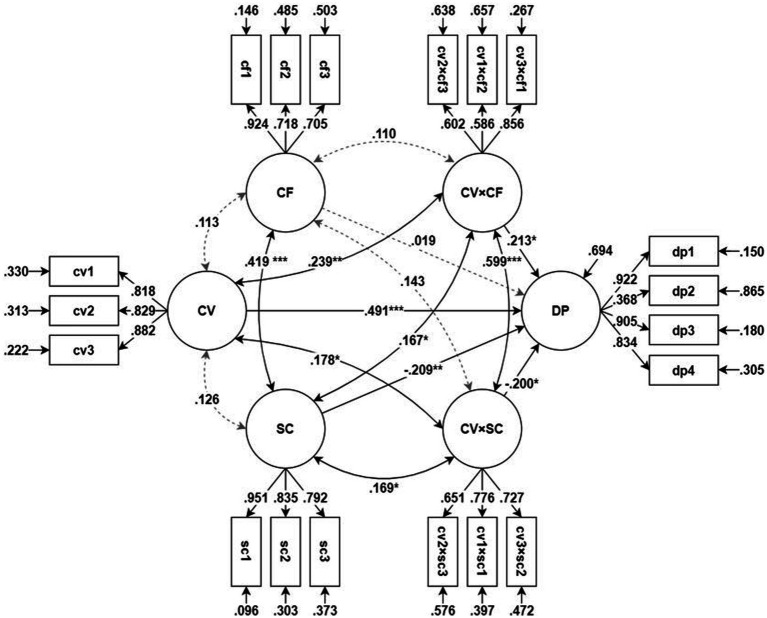
Moderation model on depression. *p < 0.05, **p < 0.01, ***p < 0.001. All factor loadings and measurement/structural error variances are statistically significant (*p* < 0.05). CV, concern/difficulty during COVID-19; DP, depression; CF, coping flexibility; SC, sense of community; CV × CF, the interaction between CV and CF (moderation of CF); CV × SC, the interaction between CV and SC (moderation of SC).

Structural equation modeling (SEM) was used to test the moderation model on depression. The model’s goodness-of-fit was determined to be satisfactory [*χ*^2^(137) = 254.132, *p* < 0.001, comparative fit index (CFI) = 0.954, Tucker–Lewis index (TLI) = 0.943, root mean square error of approximation (RMSEA) = 0.056, and standardized root mean square residual (SRMR) = 0.076]. Table 3; Figure 1 show test results for the moderation model on depression. Table 3 shows the statistical test results of the model path coefficients, while Figure 1 shows a summary of the results from testing the moderation model on depression.

**Table 3:** Testing moderation model on depression.

Model	Paths	Unstandardized coefficients (*B*)	standardized Coefficients (*β*)	Standard error	*z*
DP	CV → DP	0.626	0.491	0.084	7.448^***^
	*CF* → DP	0.028	0.019	0.098	0.289
	SC → DP	−0.183	−0.209	0.058	−3.171^**^
	CV × *CF* → DP	0.605	0.213	0.259	2.336^*^
	CV × SC → DP	−0.307	−0.200	0.139	−2.205^*^
AN	CV → AN	0.693	0.534	0.086	8.054^***^
	*CF* → AN	0.058	0.038	0.099	0.583
	SC → AN	−0.150	−0.167	0.058	−2.580^*^
	CV × *CF* → AN	0.623	0.214	0.262	2.380^*^
	CV × SC → AN	−0.335	−0.212	0.142	−2.369^*^
PTG	CV → PTG	0.411	0.188	0.123	3.349^**^
	*CF* → PTG	0.652	0.254	0.158	4.117^***^
	SC → PTG	0.638	0.422	0.091	6.970^***^
	CV × *CF* → PTG	−0.607	−0.124	0.392	−1.548
	CV × SC → PTG	0.425	0.161	0.212	2.004^*^

^*^p < 0.05, ^**^p < 0.01, ^***^p < 0.001. CV, concern/difficulty during COVID-19; DP, depression; AN, anxiety; PTG, posttraumatic growth; CF, coping flexibility; SC, sense of community; CV × CF, the interaction between CV and CF (moderation of CF); CV × SC, the interaction between CV and SC (moderation of SC).

The results showed that COVID-19-related concerns and difficulties significantly increased depression (*β* = 0.491, *z* = 7.448, *p* < 0.001); *CF* did not have a significant direct effect on depression (*β* = 0.019, *z* = 0.289, *p* = 0.772) and SOC had a significant direct effect on depression (*β* = −0.209, *z* = −3.171, *p* = 0.002). The moderating effects of *CF* (*β* = 0.213, *z* = 2.336, *p* = 0.020) and SOC (*β* = −0.200, *z* = −2.205, *p* = 0.027) were all significant. Moreover, SOC positively moderated the effects of the independent variables, whereas *CF* negatively moderated the effects of the independent variables. The explanatory power (*R*^2^) of this model for depression was 0.306.

Since the moderating effects of *CF* and SOC were significant, a simple slope test was performed to test the moderating effects. In other words, the effect size (slope) of COVID-19-related concerns and difficulties on depression was tested when the latent score of *CF* and SOC was 1, 0, or −1. The simple slope test results are shown in Table 4. The simple slope test results showed that the effects of COVID-19-related concerns and difficulties on depression increased significantly as *CF* increased, and decreased significantly as SOC increased.

**Table 4:** Result of simple slope test on depression model.

Models	Effect of CV	Latent score of moderation variables					
		Coping flexibility			Sense of community		
		−1	0	+1	–1	0	+1
DP	Slope (*B*)	0.021	0.626	1.230	0.933	0.626	0.318
	Standard error	0.284	0.084	0.260	0.164	0.084	0.161
	Wald’s *z*	0.074	7.448^***^	4.738^***^	5.685^***^	7.448^***^	1.971^*^
AN	Slope (*B*)	0.071	0.693	1.316	1.029	0.693	0.358
	Standard error	0.287	0.086	0.264	0.168	0.086	0.164
	Wald’s *z*	0.246	8.054^***^	4.989^***^	6.134^***^	8.054^***^	2.189^*^
PTG	Slope (*B*)				−0.140	0.411	0.837
	Standard Error				0.245	0.123	0.245
	Wald’s *z*				−0.056	3.349^***^	3.415^***^

^*^p < 0.05, ^**^p < 0.01, ^***^p < 0.001. CV, concern/difficulty during COVID-19; DP, depression; AN, anxiety; PTG, posttraumatic growth.

#### 2.2.4. Impact on anxiety

**Figure 2:**
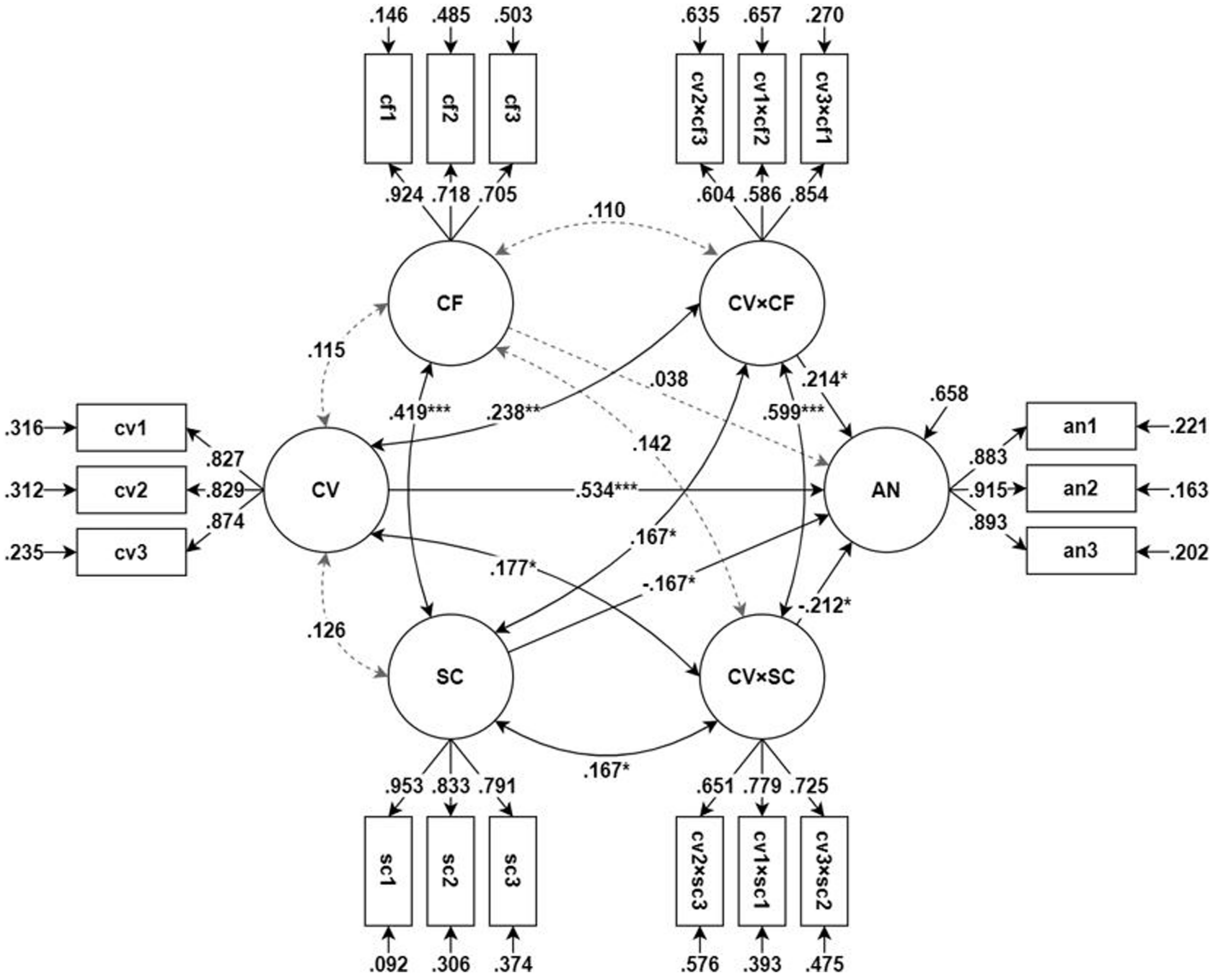
Moderation model on anxiety. *p < 0.05, **p < 0.01, ***p < 0.001. All factor loadings and measurement/structural error variances are statistically significant (p < 0.05). CV, concern/difficulty during COVID-19; AN, anxiety; CF, coping flexibility; SC, sense of community; CV × CF, interaction between CV and CF (moderation of CF); CV × SC, interaction between CV and SC (moderation of SC).

SEM was used to test the moderation model on anxiety. The model’s goodness-of-fit was determined to be satisfactory [*χ*^2^(120) = 149.909, *p* = 0.033, CFI = 0.988, TLI = 0.985, RMSEA = 0.030, SRMR = 0.043]. The test results for the moderation model on anxiety are given in Table 3; Figure 2.

COVID-19-related concerns and difficulties significantly increased anxiety (*β* = 0.534, *z* = 8.054, *p* < 0.001), while *CF* did not have a significant direct effect on anxiety (*β* = 0.038, *z* = 0.583, *p* = 0.560); SOC had a significant direct effect on anxiety (*β* = −0.167, *z* = −2.580, *p* = 0.010). The moderating effects of *CF* (*β* = 0.214, *z* = 2.380, *p* = 0.017) and SOC (*β* = −0.212, *z* = −2.369, *p* = 0.018) were significant. Furthermore, SOC positively moderated the effects of the independent variables, whereas *CF* negatively moderated the effects of the independent variables. The explanatory power (*R*^2^) of this model for anxiety was 0.342.

As the moderating effects of *CF* and SOC were significant, a simple slope test was performed to test the simple moderating effects. In other words, the effect size (slope) of COVID-19-related concerns and difficulties on anxiety was tested when the latent score of *CF* and SOC was 1, 0, or −1. Table 4 shows the simple slope test results, indicating that the effects of COVID-19-related concerns and difficulties on anxiety increased significantly as *CF* increased and decreased significantly as SOC increased.

#### 2.2.5. Effects on posttraumatic growth

**Figure 3:**
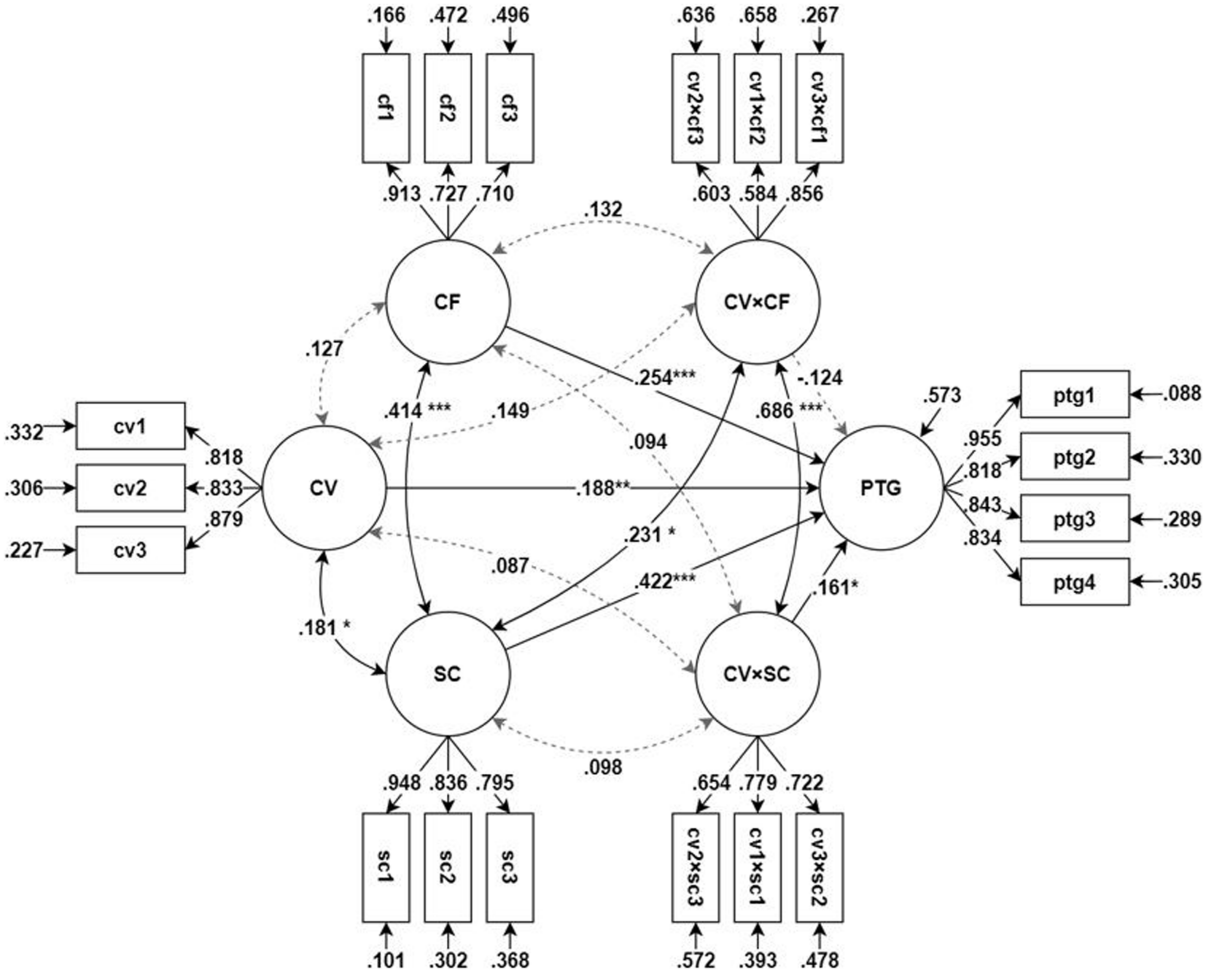
Moderation model on posttraumatic growth. *p < 0.05, **p < 0.01, ***p < 0.001. All factor loadings and measurement/structural error variances are significant statistically (*p* < 0.05). CV, concern/difficulty during COVID-19; PTG, post-traumatic growth; CF, coping flexibility; SC, sense of community; CV × CF, interaction between CV and CF (moderation of CF); CV × SC, interaction between CV and SC (moderation of SC).

SEM was used to test the moderation model on PTG. The model’s goodness-of-fit was determined to be satisfactory [*χ*^2^(137) = 189.918, *p* = 0.002, CFI = 0.981, TLI = 0.976, RMSEA = 0.038, SRMR = 0.045]. Table 3; Figure 3 show the test results for the moderation model on PTG.

COVID-19-related concerns and difficulties significantly increased PTG (*β* = 0.188, *z* = 3.349, *p* = 0.001), and both *CF* (*β* = 0.254, *z* = 4.117, *p* < 001) and SOC (*β* = 0.422, *z* = 6.970, *p* < 0.001) had a significant direct effect on PTG. The moderating effects of *CF* were not significant (*β* = −0.124, *z* = −1.548, *p* = 0.122), whereas the moderating effects of SOC were significant and positively moderated the effects of the independent variables (*β* = 0.161, *z* = 2.004, *p* = 0.045). The explanatory power (*R*^2^) of this model for PTG was 0.427.

Since the moderating effects of SOC were significant, a simple slope test was performed to test the simple moderating effects. In other words, the effect size (slope) of COVID-19-related concerns and difficulties on PTG was tested when the latent score of SOC was 1, 0, or −1. Table 4 shows the simple slope test results, indicating that the effects of COVID-19-related concerns and difficulties on PTG increased significantly as SOC increased.

## 3. Study 2

### 3.1. Materials and methods

#### 3.1.1. Participants

Among the participants in Study I, 16 participants with depression (CES-D) and anxiety (GAD-7) scores below the 50th percentile and PTG (PTGI-X) score above the 75th percentile, who consented to the interviews, participated in the study. Seven participants actually participated in the interviews. Table 5 presents information regarding the seven participants interviewed. For ethical protection, the researcher confirmed voluntary participation in the interviews and clearly explained the study objective as well as confidentiality and anonymity of data obtained through the interviews.

**Table 5:** Information regarding interview participants.

Participant	Sex	Age (years)	Primary residence^*^	Grade	Religious	Major
1	Female	20	Seoul	2	Non-Religious	Engineering
2	Female	23	Seoul	3	Non-Religious	Engineering
3	Female	21	Gyunggi-do	2	Non-Religious	Humanities/Social Sciences
4	Female	19	Gyunggi-do	1	Religious	Humanities/Social Sciences
5	Female	22	Gyunggi-do	4	Non-Religious	Education
6	Male	19	Jeolla-do	2	Non-Religious	Arts/Physical
7	Female	23	Gyeongsang-do	4	Religious	Education

^*^Primary residence during college life.

#### 3.1.2. Data collection and analysis methods

The interviews were conducted between October 4 and 8, 2021. Considering the risk of COVID-19, the interviews were conducted online via Zoom. The researcher personally transcribed the contents of each interview within 3 days from the completion of the interviews. To explore participants’ experiences during the COVID-19 pandemic, the key interview questions were constructed by referring to questionnaires used in qualitative studies on the PTG process conducted by Im (2013), Kim and Choi (2017), and Lee (2017). The participants were asked to respond to three main questions: (1) What constraints and difficulties have you experienced due to the COVID-19 pandemic? (2) How has your life changed compared to the past as a result of the COVID-19 pandemic? (3) What coping strategies or efforts did you employ to overcome the challenges caused by the COVID-19 pandemic? (Additional probing questions related to *CF* and SOC were included).

A phenomenological qualitative research method was used to analyze the interview results. Phenomenological research views the person and his/her world as inextricably linked and refrains from focusing on the world or individual as distinct entities, but explores the essence of the meaning of their interactions (Creswell, 1998). Accordingly, the purpose of phenomenological research is to find solutions to the meaning, structure, and essence of individuals’ lived experiences of a particular phenomenon (Johnson and Christensen, 2004). In this study, Moustakas’ method (Moustakas, 1994), which is a more specific phenomenological analysis technique, was used. After the interviews, the data were initially qualitatively coded by one researcher, and then continuously discussed, reviewed, and revised by a total of two researchers, including the initial coder. To ensure that the researcher’s experiences, biases, and assumptions did not influence the understanding of the phenomena of interest in this study, the researcher underwent a process of self-reflection. Additionally, the researcher consistently made efforts to view the phenomena observed in the data as new and distinct. For instance, the researcher considered the fact that they have studied the theory of PTG and participated in related research, alongside having interactions with university students who have experienced difficulties related to the COVID-19 pandemic. Subsequently, the researchers received feedback from the participants to assess whether the concepts and themes derived from the research findings accurately reflected their intentions and experiences; there were no issues identified in the analysis results.

### 3.2. Results

Based on our analysis of the interview data, 132 meaningful states and 65 themes (semantic units) were identified (Table 6). The themes were classified into 18 theme clusters for ease of interpretation, and the theme clusters were grouped into four categories: COVID-19-related constraints and difficulties and adaptation to them; coping with COVID-19-related difficulties; SOC and communal efforts; and COVID-19-related positive changes and growth.

**Table 6:** Analysis of interview data.

Category	Theme Cluster	Theme
COVID-19-related constraints and difficulties	Difficulties with college life/academics	Inconvenience of online classes / Not being able to experience the desired college life / Restrictions on practical courses / Restricted access to school facilities / Academic limitations due to financial difficulties / School seems unfamiliar
	Psychological problems	Anxious / Tense and withdrawn / Sensitive / Suppressed feelings / Feel helpless / Depressed
	Difficulties with daily/school life	Frustrated with masks / Difficulties with leisure activities / Financial difficulties at home / Restrictions on all aspects of life / Mostly indoor activities / Difficulties with going out
	Difficulties with interpersonal relationships	Difficulties with forming new relationships / Superficial relationships / Restrictions on meeting people / People’s sensitive reaction / Neglected some relationships
Coping with COVID-19 related difficulties	Coping flexibly within constrains	Leisure activities alone / Leisure activities at home / High resilience / Meeting with a small number of people while adhering to quarantine rules / Leisure activities with a few people / Changes in academics
	Finding a way forward by adapting to COVID-19	Think positively about the current situation / Find ways to improve the current situation
SOC and communal efforts	Participants’ thoughts on the SOC	Believed that they usually did not have a strong SOC for regional community and country / Increased SOC since COVID-19 / Usually had a strong SOC for country / Normally had a strong SOC / Usually experienced a sense of belonging with the school / Does not believe SOC is helpful in overcoming COVID-19 / Dissatisfaction with current quarantine rules / Personal consideration is important, not SOC / Important to adhere to quarantine rules
	Sense of solidarity/ belonging (membership)	Understand the disposition of people facing difficulties due to COVID-19 / Understand the difficult circumstances in school/community / Tolerant view of the response given by the community that they belong to
	Considerateness	Try not to be a nuisance (cause infection) to others
	Law-abiding spirit	Must adhere to quarantine rules / Good adherence to quarantine rules / Positively view people who adhere well to quarantine rules
	Responsibility/gratitude	Thankful to healthcare workers and think about something they themselves can do
COVID-19-related positive changes and growth	Enhanced coping ability	Reflection of uncomfortable feelings (not avoiding them) / Healthy coping for stress / Increased flexibility (flexible coping) / Focus on the present and find a peace of mind
	Expansion of value system and worldviews	Broader interest and awareness / Sense of value and meaning in life
	Reflective life	Self-reflection / Deeper understanding of self
	Independent life	Serious contemplation and reflection about career / Live an independent life / Focus on self and the present
	Positive changes in interpersonal relationships	Deeper relationships with family / Deeper relationships with friends / Opportunity to reevaluate relationships / Better understanding of others

#### 3.2.1. COVID-19-related Constraints and difficulties

##### 3.2.1.1. Difficulties with college life/academics

The participants were unable to enjoy a happy college life, or a “campus life.” Group activities, such as freshmen orientation and membership training (MT), could not be conducted, while participants were also deprived of the opportunity to meet classmates, upper classmen, and professors and could not obtain information regarding school life. Online classes could interfere with effective learning since facilities and equipment were not properly prepared for online classes and several restrictions were placed on practical courses, such as physical education or teaching practice. Moreover, instructors and students experienced difficulties in adapting to suddenly having to use a mouse and keyboard to communicate, instead of a pen that they were familiar with. Due to restricted access to school facilities, some students had grievances about issues such as tuition fees, while others were forced to change their academic plan due to financial problems at home.

##### 3.2.1.2. Psychological problems

The participants experienced various psychological problems due to COVID-19. They suppressed their feelings about experiencing such difficulties as everyone was undergoing the same stressful situation. As a result, they became psychologically more sensitive, while any negative news about COVID-19 made them nervous and depressed. As they spent their time only at home due to quarantine restrictions, they felt helpless and depressed. Moreover, they became anxious thinking about an uncertain future or about what other types of major events could happen, like in a disaster movie.

##### 3.2.1.3. Difficulties in daily/leisure life

Restrictions on trivial daily life events cause a certain amount of inconvenience. During the COVID-19 pandemic, college students were unable to attend and enjoy cultural facilities such as movie theaters, concerts, and sports facilities, and constantly wearing a mask outside the house was annoying and frustrating as it hampered activities. The participants felt like victims due to all the restrictions placed on various aspects of their lives. There were instances in which they experienced financial hardships due to their parents’ reduced income. They could not enjoy traveling since there was only a limited number of places they could visit. Moreover, as they spent their days only at home, they became less active, leading to poor health.

##### 3.2.1.4. Difficulties with interpersonal relationships

The pandemic hampered participants’ interpersonal relationships. As going out itself was restricted, participants could not meet people and began to experience social loneliness. They had few opportunities to meet their college classmates, and most often, they did not recognize their faces. Moreover, they regretted the fact that even when they met other college students, including classmates, they did not have the opportunity to interact with each other as they only met online for team projects due to social distancing. Some participants also experienced difficult situations in their part-time jobs, especially when customers complained at the stores they worked at, about issues such as infection control or hygiene issues.

#### 3.2.2. Coping with COVID-19-related difficulties

##### 3.2.2.1. Coping flexibly within constraints

Participants experienced difficulties and inconveniences in both their daily and college life due to certain constraints, even though they tried to cope and functioned flexibly within the enforced restrictions by meeting friends and maintaining relationships while adhering to the quarantine rules. They studied with friends using video chat programs or tried to relieve their stress through “virtual parties.” For leisure activities, participants came across as many things that they can do at home. For example, they watched YouTube videos, listened to music, or painted and drew pictures. In fact, some enjoyed their hobbies more actively than before. Some went for walks or to a coin karaoke by themselves, while others found time for reflection by writing about their life.

##### 3.2.2.2. Finding a way forward with COVID-19

The participants tried to view the pandemic situation positively. Although they could not maintain their relationships with many people, they viewed this as an opportunity to build rapport with people that they did not communicate with. Some participants felt that exercising was more effective as it was more strenuous to exercise while wearing a mask. Other participants tried to actively take advantage of the situation by discussing how to adapt, develop, and move forward with friends than think about the stressors of the COVID-19 pandemic.

#### 3.2.3. Sense of community and communal efforts

##### 3.2.3.1. Various thoughts on SOC

The participants reported varying degrees of SOC for the group, regional community, and country to which they belonged. Some participants had a strong sense of community and country, whereas other participants had a weak sense of the same. In fact, they expressed a strong SOC for their school and community clubs, and doubted if SOC could help them overcome the difficulties they experienced during COVID-19. While some expressed dissatisfaction about the government overly emphasizing SOC in enforcing quarantine rules, other participants agreed that SOC will have a positive role.

##### 3.2.3.2. Sense of solidarity/belonging (membership)

A sense of solidarity and belonging enabled participants to develop a positive mindset and awareness of others living around them. Participants considered the difficulties of family members and neighbors as their own and showed concern for those who faced financial difficulties due to COVID-19, while adopting a positive attitude toward quarantine guidelines implemented by the school or community.

##### 3.2.3.3. Considerateness

SOC involves being considerate and civil, avoiding to cause nuisance or harm to others, such as spreading the infection. For example, some participants did not engage in conversations when they went out or ordered food through delivery services, while other participants tried to wear high-quality masks to ensure that they did not spread the virus to others.

##### 3.2.3.4. Law-abiding Spirit

Owing to the strong law-abiding spirit of upholding the law and strictly following rules, Koreans filled out visitor logs when entering a building and willingly cooperated with contact tracing investigations. The participants believed that it would be helpful to cooperate with government guidelines and have a tolerant view. They also had a positive attitude toward others who adhered to the quarantine rules and tried to do the same themselves. Some participants believed that adherence to COVID-19 public health guidelines was not only appropriate but also ethical.

##### 3.2.3.5. Responsibility/gratitude

The participants were thankful to healthcare workers for their efforts to serve others during the COVID-19 pandemic. They were inspired and grateful for the efforts made by public health workers and government officials, who had worked hard to overcome the challenges associated with the pandemic. This sense of gratitude enabled them to endure and overcome inconveniences and develop a sense of purpose to help combat the pandemic.

#### 3.2.4. COVID-19-related positive changes and growth

##### 3.2.4.1. Enhanced coping ability

Even though most participants may have suffered from the stressful COVID-19 situation, they were now trying to find specific ways to relieve the stress. They managed stress by pursuing hobbies, thus taking advantage of the actual benefits of school closure. Moreover, instead of focusing on a single plan, as in the past, the participants acknowledged that they were now implementing several plans and options to factor many possibilities. They were now adopting a flexible attitude, such as being “cleverer” in coping with each situation, case-by-case, as social distancing levels changed according to the quarantine rules. One participant, who used to live a “hurry, hurry” life, meditated and took a step back to reflect, while other participants changed their perspective to accept the COVID-19 pandemic as positively as possible.

##### 3.2.4.2. Expansion of value system and worldview

As the participants examined how other countries were coping with the global impact of COVID-19, they took interest in the cultural dimensions of other countries. They appreciated international events and had the opportunity to engage in philosophical conversations with friends. Prior to the pandemic, participants admitted that they only read their curriculum books; however, they were now reading books on various subjects and also expanded their awareness by looking at things from a different perspective. The participants stated that their perception of the world had changed and they were experiencing a sense of value and meaning in life.

##### 3.2.4.3. Reflective life

As the participants spent more time alone, they had the opportunity to reflect on their past experiences and inner self. They reviewed their lifestyle and took greater interest in health, including exercise and dietary habits. The participants took a brief respite from their fast-paced life to think about their own personality or things they like. They also sorted out their feelings while reflecting on their past experiences or kept records and examined their life during the COVID-19 pandemic, to realize their self-image or the direction they desired in life.

##### 3.2.4.4. Independent life

Instead of being influenced by others’ opinions, the participants were able to reflect on their own life and pursue their own life standards. They were now able to live independently by choosing, planning, and doing what they genuinely wanted to pursue during the COVID-19 pandemic. Moreover, self-reflection helped them to take a personal inventory and think deeply about their career, making active efforts to realize their career goals and leaving multiple career options open.

##### 3.2.4.5. Positive changes in interpersonal relationships

The pandemic provided participants with an opportunity to develop strong bonds with people who were dear to them. With social distancing restricting face-to-face meetings, they could interact with only a few friends, which helped build deeper and more intimate relationships. Moreover, participants communicated better with other family members owing to spending more time with them. Some participants admitted that the pandemic gave them adequate time to focus more on important relationships and sort out unnecessary ones. Furthermore, the participants found adequate time to improve their self-awareness and build strong bonds with family and friends.

## 4. Discussion

This study tested if the effects of COVID-19-related concerns and difficulties on depression, anxiety, and PTG among Korean college students were moderated by *CF* and SOC. In-depth interviews were conducted as a qualitative research method to explore the specific details of college students’ experience during the COVID-19 pandemic.

In Study I, the hypotheses that (1) COVID-19-related concerns and difficulties would increase levels of depression, anxiety, PTG; and (2) *CF* and SOC would mitigate the impact on depression and anxiety while enhancing the impact on PTG, were tested. The hypotheses were partially supported in the study. This study showed that COVID-19-related concerns and difficulties increased Korean college students’ depression and anxiety levels significantly, leading to psychological problems. These findings are consistent with previous studies, which reported that people experienced multiple psychological problems during the COVID-19 pandemic, including anxiety, depression, fear, anger, and loneliness (Korean Society for Traumatic Stress Studies, 2020, April 7; Lee et al., 2020; Wang et al., 2020). COVID-19-related concerns and difficulties not only had a negative impact on mental well-being, but also increased PTG. Thus, while it was determined that the pandemic affected college students negatively, they could also cope with such difficulties paradoxically. These findings were consistent with a few previous studies reporting that the stress level or severity of the event could positively predict PTG or post-stress growth (Park et al., 1996; Levi and Bachar, 2019). Park and Im (2021) reported that COVID-19-related constraints and concerns did not have a significant effect on depression and anxiety; in fact, greater COVID-19-related problems (i.e., constraints and concerns) had a positive impact and actually increased commitment, with PTG facilitating such relationships. As indicated in various studies on PTG, cognitive processing must take place after a stress event and, paradoxically, such cognitive processing can be facilitated by psychological distress (Davis et al., 1998; Im and Kwon, 2013). In other words, emotional stimulation that facilitates cognitive processing is needed to achieve PTG, and COVID-19-related concerns and difficulties may have triggered such emotional stimulation.

*CF* moderated the impact of COVID-19-related concerns and difficulties on depression and anxiety; however, contrary to expectations, it amplified the impact of such disruptive emotions. Such findings were inconsistent with previous studies reporting that *CF* plays a critical role in protecting mental well-being (Bonanno et al., 2011; Ng et al., 2014; Park et al., 2015; Cheng et al., 2021). Previous studies on *CF* (Forsythe and Compas, 1987; Levenson et al., 1989; Kim et al., 1997; Cheng et al., 1999; Cheng, 2003; Troy et al., 2013) have already identified that the outcome of coping methods could vary depending on the intensity of the situation the person experiences. The finding that *CF*, which is a proposed concept that indicates the limitations of using a specific coping method, further increased depression and anxiety due to a stressful situation was inconsistent with previous studies wherein *CF* was a protective factor of mental health. With respect to how such findings could be interpreted, for individuals to have high *CF*, they need the cognitive ability to carefully identify the specific needs of a stressful situation (Neufeld, 1999). During this process, they not only identify the needs associated with the situation they face, but also assess their coping resources or behaviors. The COVID-19 pandemic was a stressful situation that lasted for more than 2 years and increased the burden of continuing cognitive efforts based on high *CF*. In addition, individuals with high *CF* attempted different coping methods during the pandemic, and if their usual coping method was not successful, they might experience relatively more subjective frustration and helplessness, which could cause depression and anxiety to increase. However, since our interpretation of the moderating effect of *CF* is not derived from relevant theories or other research findings, it cannot be definitively confirmed. For a more definitive interpretation of these research findings, future studies should examine the role of *CF* to combat a COVID-19-like situation that has a significant social impact and is experienced over a prolonged period.

SOC reduced the impact of COVID-19-related concerns and difficulties on depression and anxiety significantly, while the direct effect of SOC was also considerable. Such findings are consistent with previous studies reporting that SOC is associated with certain indicators of psychological adjustment (Pretty et al., 1994; Greenfield and Marks, 2010; Choi and Moon, 2013; Gattino et al., 2013; Rollero et al., 2014; Ryu, 2014; Cho and Kim, 2016; Choi and Park, 2018; Lee, 2018). In particular, it is noteworthy that SOC went beyond the positive effect on mental health and alleviated the impact of COVID-19-related problems on depression and anxiety. Therefore, the role of SOC in protecting mental well-being and enhancing the individual’s quality of life, which was supported in normal situations, was also supported during the pandemic. In fact, SOC can promote harmony and cooperation (Prezza et al., 2008) and provide a sense belonging (Al-Omoush et al., 2020), thereby helping one respond to the pandemic and adjust psychologically by alleviating the fear of infection. Moreover, SOC could be a key protective factor of mental health, especially during challenging situations like the COVID-19 pandemic, requiring collaborative efforts from both individuals and community.

The moderating effect of *CF* on PTG was not significant, whereas SOC had a moderating effect on the effects of COVID-19-related concerns and difficulties on PTG, and the direct effect of SOC on PTG was also significant. In other words, unlike *CF*, which was an individual-level variable, SOC was a social-level variable that protected the mental health of Korean college students and promoted PTG from negative experiences during the pandemic. During COVID-19, SOC amplified the process of achieving PTG. As suggested by Chen and Bonanno (2020) and reported in a previous study (Choi and Park, 2018), SOC was associated with resilience. Depending on how an individual behaves, thinks, and feels about people and the community, it is possible to relieve negative responses to COVID-19, such as depression and anxiety, and promote growth. By being considerate and helpful toward other members of the community, based on SOC, it is possible to promote interpersonal relationships such as accepting help from others and experiencing a deep sense of friendship. By coping together and facing problems, such as the COVID-19 pandemic, SOC can enable individuals to view the problem from a broader perspective, beyond a personal outlook.

**Figure 4:**
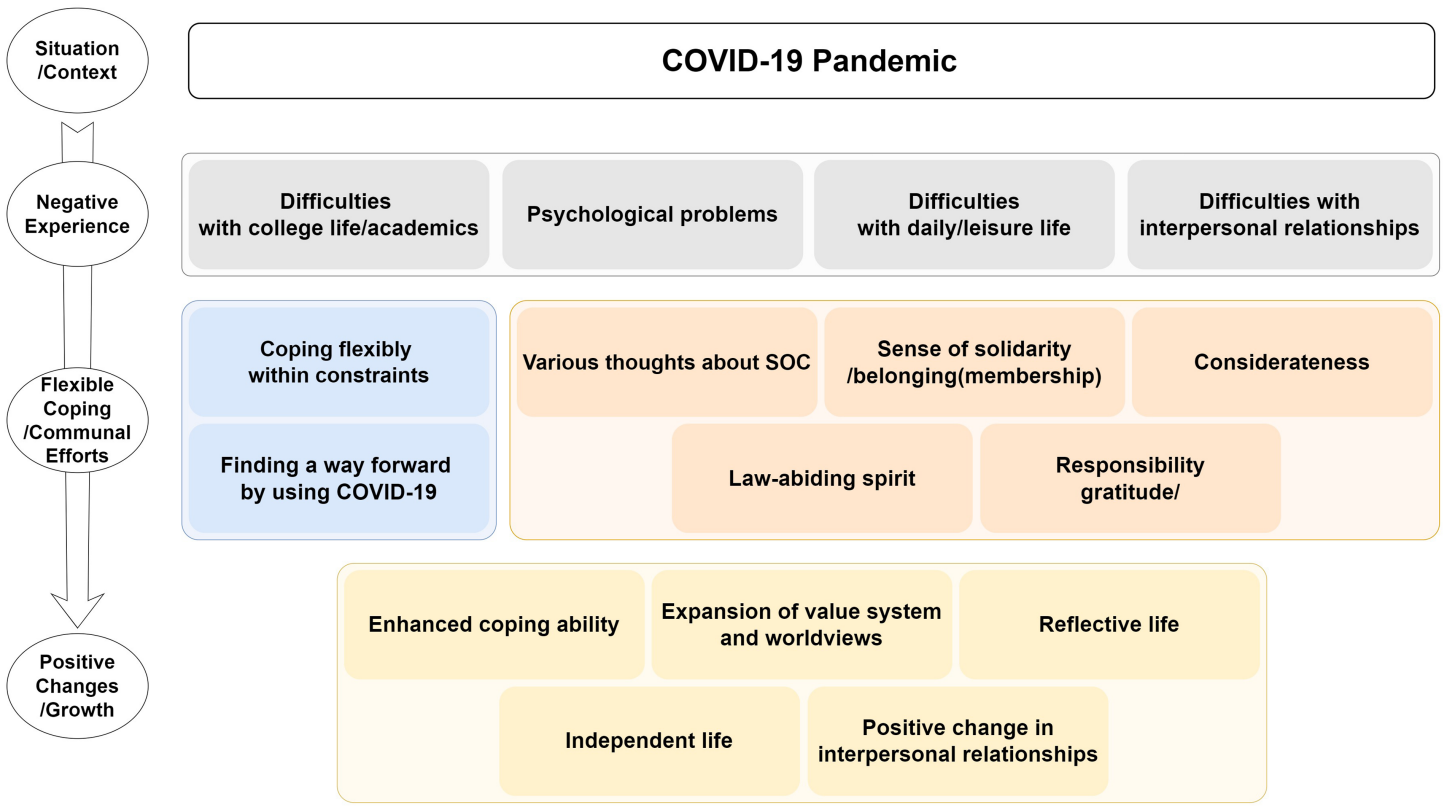
Negative experiences and adaptation, related coping and communal efforts, and positive changes and growth during the COVID-19 pandemic.

Study II was a qualitative study that aimed to further specify the findings from Study I through in-depth interviews conducted with college students from Study I who had high PTG scores. Difficulties experienced during the COVID-19 pandemic, coping with such difficulties, beliefs about SOC, and COVID-19-related positive changes or growth were extracted. Figure 4 presents a visual summary of participants’ experience with the situation.

The participants experienced various difficulties during the COVID-19 pandemic, including meeting people and maintaining relationships, college life and academics, psychological problems such as depression and tension, and restrictions on daily and leisure life. Despite many COVID-19-related restrictions, the participants coped with the situation to maintain their life and attempted to use COVID-19 to move forward. The elements of SOC that were helpful in overcoming COVID-19-related difficulties were consideration, solidarity, gratitude, and law-abiding spirit.

The COVID-19-related hardships fueled growth. Restrictions on social activities increased self-reflection and participants began to live an independent life. They also reported that the pandemic had broadened their value system and worldview, and strengthened their interpersonal relationships. The participants showed that they adapted to COVID-19-related difficulties and coped flexibly with such difficulties; in fact, they were also considerate toward their peers and others and helped them overcome these difficulties. Study I findings showed that COVID-19-related concerns and difficulties increased the effect on PTG and, for a negative event to lead to PTG, it is necessary to provide adequate emotional stimulation that can facilitate cognitive processes for the event (Davis et al., 1998; Im and Kwon, 2013). Study II findings showed that the participants continued to experience various difficulties, including psychological problems, although they had adequate emotional stimulation to reach PTG during the COVID-19 pandemic.

Korean college students’ considerate behavior and law-abiding spirit helped them overcome the difficulties associated with COVID-19. In fact, such compassion and upright behavior are related to social responsibility or reciprocity, which is a practical attitude for maintaining a community. Membership, which McMillan and Chavis (1986) proposed as an element of SOC, is associated with a sense of ethics and responsibility toward the community (social responsibility), while shared emotional connection is associated with awareness that spreads as direct action toward others (reciprocity) (Kim, 2000).

This study has certain limitations. First, the gender ratio and religious status of the samples were not balanced. In Study I, there were more than three times as many females as males, while in Study II, only one of seven participants was a male. The content of concerns or difficulties experienced during the COVID-19 pandemic may vary between males and females due to cultural differences. Moreover, the religious status of the samples was asymmetrical. Considering that an individual cannot control the characteristic of COVID-19, the possibility that religious coping may influence mental health or growth cannot be dismissed. In particular, PTG has various psychometric attributes depending on religious status (Im, 2015; Sim et al., 2021); thus, religious status is an important issue with respect to generalization of the model for PTG. Second, various confounding variables were not considered during the modeling process. Since the COVID-19 pandemic has been ongoing over a prolonged period, various factors may have had a significant psychological impact on the population. Unevenness of samples is a problem related to not considering categorical confounding variables. Accordingly, future studies should consider various confounding variables, including collection of COVID-19-related information, residential types, trust in government or health authorities, tolerance of uncertainty, infection status of self or others, and faith in religion. Third, there were issues concerning the interpretation of the results. in both the quantitative and qualitative analysis stages. Regarding the quantitative analysis results, there was a somewhat limited theoretical basis for the moderating effects. Specifically, further research is needed to explore the moderating effect of *CF*, which was contrary to the hypothesized direction. The proposed interpretations regarding the moderating effect of *CF* in this study should be cautiously accepted, as they are based solely on our findings. Additionally, there was a somewhat limited theoretical and empirical basis for the relationship between SOC and PTG, although this study was conducted from an exploratory perspective. More robust theoretical support and future research are needed to understand the mechanisms by which SOC enhances PTG. Furthermore, the small number of participants in Study II (seven participants) may have limited the generalizability of our findings.

Despite these limitations, the present study is important as it used modeling to test the mental well-being and growth experience of Korean college students during the COVID-19 pandemic and qualitatively examined their experiences. Especially, an additional qualitative study that encompassed more refined and rich information about the experiences of Korean college students during the COVID-19 pandemic, which cannot be obtained by structured measures and statistical analysis, was obtained. The findings showed that during a disaster situation, namely the COVID-19 pandemic, not only intrapersonal factors but also sociocultural factors such as SOC played a crucial role in protecting mental well-being. Korean college students experienced various difficulties during the COVID-19 pandemic; paradoxically, such experiences helped them grow as individuals. In other words, an integrative perspective that distinguishes between the dimensions of emotional distress and psychological growth can help to understand an event and how an individual experiences it. During difficult situations such as COVID-19, having an integrative perspective about what is difficult and distressful and how new opportunities for growth can be found and achieved is important. For example, in the psychological intervention related to trauma, the clients would be able to experience the process of handling their circumstances and reassessing them to gain a new perspective by understanding trauma-related emotions (Baranowsky et al., 2011). For the practical use of these findings, future studies should investigate whether psychological interventions that promote SOC help patients’ PTG experiences, especially in unique stressful situations such as COVID-19.

## 5. Conclusion

South Korea is recognized as one of the prominent nations that implemented successful containment measures during the COVID-19 pandemic. Korean college students experienced significant discomfort and distress due to the pandemic; however, it was found that greater levels of distress were associated with higher levels of PTG. The importance of SOC in reducing the negative impact of the pandemic and promoting growth has been highlighted. There is a substantial possibility of future outbreaks of such infectious disease disasters. Therefore, it is crucial for the government and educational institutions to emphasize through campaigns the significance of social cooperation and responsibility in safeguarding individuals’ lives and freedoms, and to prepare them to effectively cope with disaster situations.

## Data availability statement

The raw data supporting the conclusions of this article will be made available by the authors, without undue reservation.

## Ethics statement

The studies involving human participants were reviewed and approved by Hallym Institutional Review Board (HIRB-2021-047-1-R). The patients/participants provided their written informed consent to participate in this study.

## Author contributions

J-CS and S-YI participated in conceptualization, data collection, and statistical analysis. J-CS and S-YI wrote the first draft on Introduction, Discussion, Method, and Results. All authors reviewed and edited the draft together, and approved the final draft.

## Funding

This research was supported by the Hallym University Research Fund, 2022 (HRF-202203-009).

## Conflict of interest

The authors declare that the research was conducted in the absence of any commercial or financial relationships that could be construed as a potential conflict of interest.

## Publisher’s note

All claims expressed in this article are solely those of the authors and do not necessarily represent those of their affiliated organizations, or those of the publisher, the editors and the reviewers. Any product that may be evaluated in this article, or claim that may be made by its manufacturer, is not guaranteed or endorsed by the publisher.

## Footnotes

^1^ http://www.phqscreeners.com/select-screener
